# Behavioral Ethics Ecologies of Human-Artificial Intelligence Systems

**DOI:** 10.3390/bs12040103

**Published:** 2022-04-11

**Authors:** Stephen Fox

**Affiliations:** VTT Technical Research Centre of Finland, FI-02150 Espoo, Finland; stephen.fox@vtt.fi; Tel.: +358-40-747-8801

**Keywords:** artificial intelligence (AI), behavioral ecology, behavioral ethics, diagnostic systems, function, gait analysis, human–AI systems, mechanism, ontogeny, phylogeny, situated entropy, vehicle navigation

## Abstract

Historically, evolution of behaviors often took place in environments that changed little over millennia. By contrast, today, rapid changes to behaviors and environments come from the introduction of artificial intelligence (AI) and the infrastructures that facilitate its application. Behavioral ethics is concerned with how interactions between individuals and their environments can lead people to questionable decisions and dubious actions. For example, interactions between an individual’s self-regulatory resource depletion and organizational pressure to take non-ethical actions. In this paper, four fundamental questions of behavioral ecology are applied to analyze human behavioral ethics in human–AI systems. These four questions are concerned with assessing the function of behavioral traits, how behavioral traits evolve in populations, what are the mechanisms of behavioral traits, and how they can differ among different individuals. These four fundamental behavioral ecology questions are applied in analysis of human behavioral ethics in human–AI systems. This is achieved through reference to vehicle navigation systems and healthcare diagnostic systems, which are enabled by AI. Overall, the paper provides two main contributions. First, behavioral ecology analysis of behavioral ethics. Second, application of behavioral ecology questions to identify opportunities and challenges for ethical human–AI systems.

## 1. Introduction

Behavioral ethics is concerned with what is done, rather than the normative ethics of what should be done. Behavioral ethics addresses the potential for good people to make questionable decisions and take dubious actions [1,2,3]. Behavioral ethics considers interactions between moral motivation and ethical temptation, which depend on combinations of variables that can come together differently in different situations [4,5,6]. These include, for example, an individual’s self-regulatory resource depletion and organizational pressure to take non-ethical actions [7,8]. Having the moral motivation to resist ethical temptation can be a struggle [9], in which non-ethical impulses can override moral reflection [10], sometimes with disastrous consequences for individuals and organizations [3]. Organizational pressure can arise from conflicts between organizational practices, such as performance reporting and performance rewarding [11]. Individuals who identify strongly with an organization can go along with organizational pressure to take non-ethical action [12,13], especially if their self-regulatory resources are depleted [14], for example from overwork for the organization [15] and/or time pressures [16,17]. Fundamentally, behavioral ethics is concerned with how interactions between individuals and their environments can lead to questionable decisions and dubious actions.

All aspects of human–environment interactions are encompassed within the field of human ecology [18]. With more specificity, cultural ecology encompasses human adaptations to social and physical environments [19]. With further specificity, human behavioral ecology addresses the same questions as behavioral ecologists when studying other species [20]. Four fundamental questions in behavioral ecology are those raised by Niko Tinbergen. Two of the questions are evolutionary as follows: what are the ecological fitness functions of behavioral traits (function)? and what is the behavioral trait’s evolutionary history in a population (phylogeny)? Two of the questions are proximate as follows: what is the structure of a behavioral trait (mechanism)? and how has the behavioral trait developed in an individual (ontogeny) [21]? Hitherto, the relevance of behavioral ecology to human ethical behavior has been recognized [22], but there has not been a behavioral ecology analysis of ethical human-artificial intelligence systems. This is despite fundamental questions in behavioral ecology being applicable to nonliving as well as living systems [23].

This gap in the literature is addressed in the remaining sections. Next, in Section 2, human–AI systems’ behavioral traits are analyzed in terms of function. In Section 3, they are analyzed in terms of phylogeny. In Section 4, they are analyzed in terms of mechanism. In Section 5, they are analyzed in terms of ontogeny. In Section 6, the four behavioral ecology questions are applied together to structure behavioral ethics analysis of a human–AI system. This is achieved with the examples of vehicle navigation systems and healthcare diagnostic systems. In conclusion, principal contributions are stated and directions for future research are proposed in Section 7. Overall, the behavioral ecology analysis identifies opportunities and challenges for ethical human–AI systems. These include the dependence of human–AI systems on ideal environmental conditions to reduce ethical stress, and the need for policy-making to encompass the multitude of environmental factors that can affect human–AI systems.

## 2. Function

The function of behavioral traits in enabling survival can be considered in terms of ecological fitness. The closer the fit between a human behavioral trait and the current environment in which the person intends to survive, the less information the person will be lacking about how to survive, the less physical disorder there will be in the person’s actions to survive in the environment, and the less energy the person will consume unproductively in actions to survive. Hence, when there is a good fit between a person’s behavioral traits and the environment, the person can survive with least action and have energy free for other actions [24,25,26]. In such situations, a person can maintain internal stability by internal regulation through homeostasis [27].

Consider, for example, the demanding working lives of truck drivers. Typically, truck drivers are under time pressure, but this could be reduced to some extent if a truck driver has the behavioral trait of excellent navigation skills. In particular, a truck driver who has the behavioral trait of excellent navigation skills can experience low situated entropy [24,25]. This is because such a truck driver does not experience information theoretic entropy from route information uncertainty. Consequently, the truck driver travels to delivery destinations directly, and so does not experience the statistical mechanics entropy of the physical disorganization involved in driving incorrect routes. As the truck driver does not drive incorrect routes, the truck driver is not under additional time pressure and may have time to take at least some rest breaks that include eating properly. Moreover, the truck driver does not experience thermodynamic entropy, which would be entailed in the physical disorganization of driving incorrect routes due to lack of route information. Rather, the truck driver survives with least action and can have some energy free for other actions, such as recreational activities that can facilitate sleeping well, which together with workday rest breaks, can contribute to maintaining balance between energy demands and energy supply.

By contrast, the worse the fit between a behavioral trait and the environment in which the person intends to survive, the more information a person will be lacking about how to survive. This can increase physical disorder in the person’s actions taken to try to survive in the environment, and increase the energy the person will expend unproductively in actions taken to try to survive. Hence, when there is a bad fit between a person’ behavioral trait and the environment, the person cannot survive with least action and cannot have much energy free for other actions. In such situations, a person may not be able to maintain internal stability by internal regulation through homeostasis. Rather, the person can experience allostatic overload. This can happen when internal regulatory work increases to the point where energy demand exceeds energy supply and a person is depleted of resources needed to function well. This can lead to altered activity in brain areas involved in law-abiding and moral behavior. Specifically, severe stress decreases activity within brain areas that support some of the highest forms of contextual integration, leads to top-down collapse of higher goals, and the favoring of short-term aims [28,29].

Consider, for example, a truck driver who does not have good navigation skills and so experiences high situated entropy [24,25]. Such a truck driver is lacking in correct route information and so does experience information-theoretic entropy from information uncertainty. Consequently, the truck driver does not travel to delivery destinations directly, and so does experience the statistical mechanics entropy of the physical disorder entailed in driving incorrect routes. As the truck driver is often driving incorrect routes, the truck driver is under additional time pressure and does not have any time to take rest breaks that include eating properly. Furthermore, as time pressure increases, the truck driver can experience increasing ethical temptation to drive through traffic lights as they are turning from orange to red. Every time the truck driver resists the temptation to drive through such traffic lights, the truck driver has to exert self-regulatory control that can become depleted.

In addition, the truck driver experiences thermodynamic entropy. Before getting into the truck, depending on previous food intake, the driver can have thermodynamic free energy available for doing useful work. However, once allocated to truck driving without correct route information, the truck driver’s energy changes from being potentially useful to being practically useless. This is because the truck driver’s energy is allocated to being lost amidst thermodynamic entropy entailed in the physical disorganization of driving incorrect routes due to lack of correct route information. Hence, the truck driver does not have energy free for other actions, such as recreational activities that can facilitate sleeping well. Thus, the truck driver who does not have good navigation skills may be less likely to maintain balance between energy supply and energy demands than a truck driver who does have good navigation skills.

As summarized in Table 1 below, as well as being under time pressure and being depleted, the truck driver may feel extreme survival pressure if a long track record of poor delivery performance has placed the truck driver under threat of dismissal from the last haulage company that will provide employment. In such a situation, the truck driver may suffer from chronic stress because of loss of resources [30] due to erratic employment in the past, and from chronic anxiety about how to survive in the future [31]. In such a situation, there can be increased potential for questionable decisions and dubious actions. For example, for the truck driver to continue to drive while feeling sleepy [32].

From the perspective of behavioral ethics, when the same truck driver is not under so much time pressure, is not depleted, and is not under imminent survival threat, the same truck driver would stop as traffic lights turn from green to orange and would not continue to drive when starting to feel sleepy. This could happen if, amidst a severe shortage of truck drivers [33], haulage companies could install new AI-support navigation systems into trucks that could improve the delivery performance of truck drivers [34]. Thus, although the human truck driver alone may have the behavioral trait of not having navigation skills, the same human in a human–AI system could have the behavioral trait of having navigation skills. This could lead to reduced situated entropy, i.e., reduced thermodynamic entropy because of reduced statistical mechanics entropy due to reduced information-theoretic entropy. Hence, the ecological fitness function of a behavioral trait is high when situated entropy arising from that trait in an environment is low.

## 3. Phylogeny

Hitherto, the evolution of behavioral traits in populations may have taken place over many generations within natural environments that changed little over millennia. For example, humans developed navigation skills, which are useful today, when we were finding our way as hunter-gathers [35,36,37]. Although human capabilities evolved through many millennia [38,39], we are now trying to survive in environments that can change rapidly at least partially because of human ecosystem engineering [40,41,42,43] and bring an increasing variety of survival threats such as unemployment [44,45] and health challenges [46,47]. Moreover, human ecosystem engineering can reduce quickly human capabilities that evolved over millennia. For example, Internet-enabled navigation systems can reduce human navigation skills [48].

The development of vehicle–infrastructure integration for human–AI systems is an example of rapid widespread ecosystem engineering. This involves the re-engineering of public roads into so called smart roads that can provide “cooperative infrastructure” for vehicles that have autonomous functionality. This involves a range of costly engineering activities, such as the installation of V2X (vehicle-to-everything) WiFi. In addition, sensors are being developed to be integrated into road surfaces that can be used to inform communication to autonomous vehicles by new types of smart road signage. The economic viability of engineering work settings depends on the number of operations over which the high financial costs can be spread. Such financial costs may perhaps be economically viable over main roads with a high frequency of vehicles, but are prohibitively expensive for low-frequency roads and for off-road locations [49,50,51,52]. Accordingly, the extent of vehicle functioning that is technically feasible can change as a truck is driven on different types of roads with different levels of cooperative infrastructure.

As summarized in Table 2 below, this example illustrates that the series of evolutionary steps in a population (i.e., phylogeny) for behavioral traits of human–AI systems can involve natural evolution over millennia combined with increasing rapid technological evolution over centuries, decades, and years. For example, human navigation capabilities have evolved over millennia, road networks have evolved over centuries, trucks have evolved over decades, and cooperative infrastructures have evolved over years.

However, the extent of each trait component can be dependent on situation-specific variables, not least environmental conditions that can limit the use of technological components. For example, erratic Internet coverage can make some AI operations erratic [53]. In addition, unfavorable weather can limit the use of technological components. For example, all technological components may function in a fully cooperative road infrastructure in favorable weather conditions, but extreme weather events can flood roads, stop trucks, and prevent cooperative infrastructure from functioning. Occurrences of such unfavorable weather conditions are increasing [54,55]. Accordingly, the function of human–AI navigation systems is not robust from an evolutionary perspective. That is, the function of human–AI navigation systems is not persistent under environmental perturbations [56,57]. Rather, the function of human–AI navigation systems is only fully operational in ideal conditions. Consequently, it can only be relied upon in ideal environmental conditions to reduce information uncertainty that could otherwise arise from poor route information that would lead to the physical disorder of driving incorrect routes with consequent unproductive energy expenditure. In summary, situated entropy in human–AI systems is dynamic as truck drivers and their vehicles pass through different environmental conditions. Thus, human–AI systems can only be relied upon in ideal conditions to reduce situated entropy, which would reduce the human truck driver’s time pressures and depletion. Hence, human–AI systems can only be relied upon in ideal conditions to reduce the potential for questionable decisions and dubious actions, such as continuing to drive while feeling sleepy.

## 4. Mechanism

Human navigational skills are founded on wayfinding involving memory, perception, and attention [58]. Whereas navigating involves following a preset route, wayfinding involves the ability to create a novel route that is based on understanding a wider frame of reference than a preset route [59,60]. Wayfinding involves creating novel routes through changing situations by making non-conscious reference to cognitive maps and conscious reference to waypoints [35,36]. Here, cognitive maps are mental representations of spatial relations [61]. Waypoints can be physical and natural, such as desert oases and rocky outcrops. Waypoints can be physical and human-made, such as beacons and buoys. Waypoints can be digital and human-made, such as landmarks in digital maps [62].

Human wayfinding skills evolved when we were hunter gatherers [63,64]. Human wayfinding can require dynamic cognitive activity [65]. In particular, dynamic embodied cognition [66]. That is, cognition that depends on sensory inputs brought by and processed by the physical body that shapes prior beliefs and action outputs [67]. Different memory capacities can affect different individuals’ formulation of cognitive maps [68], which can be held within different individuals’ different embodied cognitive architectures [69,70]. Human skills that have been evolved over millennia of wayfinding can be quickly reduced by current human ecosystem engineering, such as global Internet-enabled navigation systems [48]. This can be due to such systems reducing the use of memory, perception, attention, and cognitive diligence that have hitherto been essential to human wayfinding [64,71]. Thus, different people can have different navigation skills, and their navigation skills can change over time.

Accordingly, if the AI components of a human-AI systems for truck navigation are not available, for example due to lack of Internet access or extreme weather, some truck drivers will sometimes get lost rather than getting straight to delivery destinations. This may be because truck drivers are not able to minimize disparities between route assumptions based on prior beliefs, individual waypoints, and overall spatial structures. There can be many opportunities for getting lost when human wayfinding skills are poor and AI-enabled navigation support is not available. For example, getting lost may be more likely when spatial structures cannot be seen as a whole. Moreover, getting lost may be more likely when an individual’s perception of the spatial structure based on prior beliefs does not correspond to the actual structure. In addition, individuals can get lost if they try to recall and to apply a specific route instead of developing a mental spatial representation by inferring location on the move. This can happen even in structured environments such as inside buildings [72], where the final phase of a delivery may need to be made. Accordingly, the human mechanism and the AI mechanism within a human–AI system may be complementary in some situations, but have negative unintended interactions in the longer term.

Thus, it cannot be assumed that a human–AI truck navigation system will eliminate information uncertainty, physical disorder, and unproductive energy expenditure involved in getting lost. Rather, situated entropy in human-AI systems is dynamic. Hence, as summarized in Table 3 below, it cannot be assumed that a human-AI truck navigation system will always prevent human truck drivers coming under time pressure and becoming sufficiently depleted to make questionable decisions and take dubious actions. Rather, there can be many scenarios where ethical behavior is at risk. Accordingly, the mechanism of a human-AI truck navigation system should include additional components to support behavioral ethics. For example, automated messages could be provided for encouraging ethical actions and reminding about ethical actions. These can be considered as cues to support moral motivation [7,73]. Such messages do not have to be dependent on Internet access because they can be communicated via text messages [74,75]. Messages can encourage ethical actions indirectly, for example, by encouraging taking the necessary number and duration of rest breaks in order to reduce the potential for truck drivers becoming depleted. Messages can encourage ethical actions directly if they are related to a performance appraisal methodology that rewards ethical behavior [73]. Messages reminding about ethical actions can be related to reminding truck drivers not to drive while feeling sleepy, because truck driver sleepiness is a major cause of road accidents [76]. All messages should be in accordance with a policy that addresses the potential for productivity incentives to lead unintentionally to unethical actions [8,77].

Thus, while the natural mechanism for human navigation has evolved over millennia, the mechanism of ethical human-AI systems for vehicle navigation also needs to encompass rapidly evolving AI for vehicle navigation. In addition, it needs to include rapidly evolving AI to provide encouragement and reminders for ethical behavior, and a management policy that is carefully formulated by and overseen by humans to facilitate intended positive ethical outcomes and prevent negative unintended ethical consequences. The management policy should involve humans having the capability to oversee the overall activity of the system in terms of its relation to laws, regulations, and standards [78]. Moreover, it should include human intervention during the monitoring of the system’s operation, and have capacity for human intervention in every decision cycle of the system. Such management policies should be documented for impartial external audit [79].

## 5. Ontogeny

How a behavioral trait develops in an individual can be affected by experience and personality. Consider, for example, the different truck drivers as summarized in Table 4. Two of them have many years of experience of navigating successfully on traditional road infrastructure. Neither has used digital navigation systems. One has not used them only because of not having needed to do so. The other has not used them because of suspicion of AI. For example, people can have concerns about AI and robotics taking jobs [80]. This could be based on the truck driver believing the use of AI would include AI learning to enable full truck automation and full truck driver unemployment in the near future [81]. At the same time, some truck drivers could have wider concerns such as AI and robotics developing dangerous superintelligence, harboring malicious intrinsic motivations, and enacting unfavorable intentions [82,83,84,85]. Hence, some people can be reluctant to participate in human-AI systems [86]. The second truck driver is prone to anxiety. The other two truck drivers are digital natives [87]. Neither of them has experience of navigating successfully without digital navigation support. One of them has not tried navigating without digital support because of not having needed to do so. The other has not tried because of suspicion of traditional methods of navigation. The fourth truck driver is prone to anxiety. Personality types with a propensity for anxiety have been associated with reluctance to use new technology [88] and with truck driver accidents [89].

Human choices are often not based on objective evaluation of utility. Rather, choices can be influenced by numerous biases and include over-emphasizing potential outcomes that are extreme but unlikely [31,90]. As summarized in Table 4 below, different experience and personality can lead to some people wanting to use new technology and other people not wanting to use the same new technology. These behaviors can be described in terms of approaching and avoiding [91,92]. In particular, environments can be perceived to carry varying degrees of danger. Passive avoidance can arise in environments where the extent of danger is uncertain. Especially for people who have a general propensity for anxiety, avoidance can be accompanied by chronically high levels of anxiety [93,94,95]. Chronic anxiety can lead to chronic stress [96,97,98]. This has serious implications for ethical behavior as severe stress can decrease activity within brain areas involved in law-abiding and moral behavior [28,29].

## 6. Behavioral Ecology Analysis of Ethical Stress in Human-AI Systems

### 6.1. Overview

**Figure 1:**
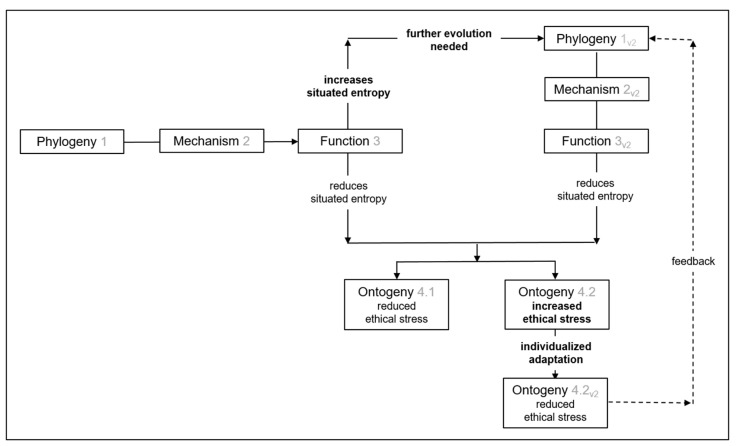
Behavioral ecology analysis of human-AI systems. Iterations of system evolution (phylogeny) and individual adaptations (ontogeny) of system mechanism are needed in order to minimize situated entropy from system function that can cause ethical stress.

An overview is provided in Figure 1 of the behavioral ecology analysis of ethical stress in human-AI systems. It is appropriate to place situated entropy at the center of analysis and development of ethical human-AI systems because as well as entropy having a determining influence over human stress [28,31,96], entropy is a fundamental concept in computer science and its applications [99,100,101,102]. In Figure 1, phylogeny (1) refers to the evolution of mechanism (2) of a human-AI system before its introduction. Function (3) refers to function at introduction, which may lead to reduced situated entropy and related ethical stress in some situations, but increased situated entropy and related ethical stress in other situations. Phylogeny (1_v2_) refers to evolution after initial introduction that leads to an adapted mechanism (2_v2_), which provides adapted function (3_v2_) with increased potential to reduce situated entropy. Ontogeny (4.1) refers to the ontogeny of one person who experiences reduced situated entropy and related ethical stress. Ontogeny (4.2) refers to the ontogeny of another person who experiences increased situated entropy and related ethical stress. Ontogeny (4.2_v2_) refers to the same person after individual adaptation of the human-AI system leads to the person experiencing reduced situated entropy and related ethical stress. Feedback refers to the potential for individual adaptations informing further phylogeny.

### 6.2. AI Navigation Support for Human Truck Drivers

AI-enabled fully automated trucks are a goal for some road freight companies. However, this corporate goal is hindered by the need for human truck drivers to take care of the so-called first mile and last mile of deliveries where there is too much task variation for AI to deal with [103,104]. Despite the continued importance of human truck drivers, current approaches to AI implementation have led to the working lives of truck drivers becoming what has been described as a dystopian nightmare. This involves surveillance of truck drivers to such an extent that even their eye movements are monitored [105]. Accordingly, new approaches are needed for AI implementations in road freight.

As summarized in Table 1, human stress that can undermine behavioral ethics can be low when function is well-matched to an environment. This is because there is low information uncertainty, low physical disorder, and low unproductive energy consumption. Hence, there is low situated entropy. However, as summarized in Table 2, human-AI system phylogeny can involve interrelated fitness components that evolve over very different time scales, and can have very different levels of distribution. Moreover, human-AI system phylogeny can lead to a behavioral trait not being robust amidst environmental perturbations. Hence, there can be many situations in which human-AI systems will not reduce situated entropy that brings ethical stress. Thus, while human-AI systems can introduce opportunities for reducing situated entropy, they can also introduce new challenges. In particular, human navigation skills can be undermined by frequent use of AI-enabled navigation systems [48], but AI-enabled navigation systems cannot be relied upon in all situations. Accordingly, human-AI navigation systems need to be situated within wider efforts that can reduce situated entropy. For example, the replacement of hundreds of separate delivery locations with common collection locations [106]. Furthermore, as summarized in Table 3, the human-AI system mechanism needs to include additional components within a management policy, for example, that limits the potential for productivity incentives to lead unintentionally to unethical actions. However, this could increase the complexity and the risk of failure of a human-AI system. Accordingly, system design for high reliability is required [107]. As summarized in Table 4, it is important that ontogeny can include individualized adaptation of human-AI systems in order to mitigate against the potential for individual differences in experience and personality to lead to chronic stress that can undermine human moral motivation. However, this can further increase complexity, which it may not be possible to offset completely with system design for high reliability [108]. An overall summary of opportunities and challenges for human-AI truck navigation systems is shown in Table 5.

### 6.3. AI Diagnostic Support for Human Healthcare Providers

Human-AI navigation systems provide quite straightforward examples of moral dilemmas, such as whether or not to drive through traffic lights as they turn from orange to red, and whether or not to continue to drive when feeling sleepy. However, there are many other potential applications of human-AI systems where moral dilemmas are less straightforward. Consider, for example, a human-AI system for planning recovery pathways for functional disorders, i.e., for medical conditions without complete medical explanation that impair normal functioning of bodily processes [109]. Here, moral dilemmas arise for healthcare organizations and their personnel from the challenge of deciding where to allocate and where not to allocate finite healthcare resources at the tax payers’ expense. In particular, lack of complete medical explanation for functional disorders can lead to concerns that they are actually factitious disorders or malingering [110]. Factitious disorder, which has also been called Munchausen Syndrome, involves people behaving as if they have illnesses by deliberately producing, feigning, or exaggerating symptoms [111]. This is different to malingering, which involves deliberate effort to simulate illness in order to get out of obligations and/or to obtain benefits [112]. Accordingly, there can be stigma against patients with functional disorders that presents obstacles to diagnosis and treatment. It has been argued that symptoms can be misunderstood or dismissed because of stigma. Moreover, it has been argued that stigma exacerbates the suffering of patients, and can result in poor clinical management involving prolonged use of healthcare resources [113]. Thus, new healthcare systems are needed that can provide better diagnosis and better recovery pathways for people suffering with functional disorders [114].

New investments in human resources are needed for new healthcare systems. In addition, artificial intelligence can contribute to new healthcare systems by carrying out automated analyses of healthcare study results, such as scans, and by analyzing patterns over a series of results. For example, gait problems are a feature of some functional disorders. Gait encompasses walking, running, and other means of natural locomotion combined with posture. Gait analysis includes the measurement of multiple parameters from which conclusions can be drawn about health [115]. Gait analysis could contribute to distinguishing between functional disorders, factitious disorders, and malingering, because gait involves complex natural processes that are difficult to fake consistently. Hence, gait analyses are used for security as well as for healthcare [116]. Some gait patterns are found frequently among patients with functional disorders. These include excessive gait slowness, knee buckling, and astasia-abasia, which refers to the inability to either stand or walk in a normal manner.

Regardless of what initiates functional symptoms, they can be perpetuated by phobic avoidance and affective disorders [117]. Accordingly, it is important that gait analysis reports do not contribute to phobic avoidance and affective disorders, but rather contribute to a care pathway for patient recovery [118]. For example, gait analysis reports can contribute to evaluation of patients’ complex disorder status, treatment, rehabilitation, and recovery [119]. In practical terms, this can involve important outcomes such as preventing wheelchair dependency [120]. A range of artificial intelligence techniques have been applied to gait analysis. These have been found to have potential for making positive contributions to detecting disorders and differentiating between disorders [121,122,123,124,125], including detecting affective disorders [116,126,127,128,129].

However, there remain many challenges in the deployment of AI for gait analyses. Notably, there has been little progress in making AI-enabled gait analyses understandable for patients and healthcare providers [130]. Hence, both can experience much information uncertainty about gait analyses. As summarized in Table 6 below, if the human-AI system does have the function of reliable and explainable gait analysis, it can reduce the patient’s situated entropy in the patient’s healthcare environment. However, this depends upon the patient agreeing with the AI-enabled gait analysis, and patient acceptance of a diagnosis can depend upon patients’ preconceptions rather than the content of diagnoses [131,132]. If the patient does not agree, the patient can experience information uncertainty, physical disorder, and unproductive energy expenditure related to trying to find alternative treatment options, all of which can lead to stress that can worsen the patient’s condition. However, even if the patient does not agree with the diagnosis, the healthcare provider can experience reduced situated entropy about the allocation of treatment resources. This is because patients who do not agree with a diagnosis are less likely to respond to treatment, and resources have to be allocated appropriately in the healthcare provision environment of scarce resources [133]. Ongoing evolution of technological components has the potential to improve diagnoses. This could lead to AI-based diagnosis based on advanced technologies being seen as being more reliable than human diagnosis. However, AI-enabled gait analysis will only be robust in an environment where the recording of the patient’s gait, for example with sensors and cameras, is ideal for their operation. At the same time, the environment must be acceptable to the patient. In addition, the mechanism of the human-AI systems can involve many software and hardware components in recording and analysis that can be difficult to combine. Moreover, patients need to be comfortable with having their gait recorded. Thus, the reliable function of objective gait analysis can be difficult to achieve even in ideal environments.

Furthermore, AI-enabled gait analysis cannot be reliable if the patient and/or the healthcare provider’s human expert are impelled to avoid AI due to personal factors such as experience and personality. In such situations, the patient could experience anxiety when interacting with AI that could unintentionally alter gait [134]. In addition, if the healthcare provider’s human expert does not trust AI [135], the expert may not base treatment recommendations on the AI-enabled gait analysis. This can be because the AI has a so-called black box model within which inputs and outputs can be seen, but the processes and workings in between them cannot be seen. New methods are being researched to obtain information from AI systems in order to generate explanations for their outputs. However, these methods have limitations such as only providing details relevant for a single decision, rather than providing underlying rationale or causality [136]. Accordingly, there is also research being carried out to develop less complex AI models. However, there are few working examples of such models in 2022 [137]. Accordingly, healthcare practitioners may be more suspicious than trusting of AI-enabled diagnoses.

In terms of ontogeny, both the patient’s and the expert’s interaction with the AI may evolve so that either or both may become more or less anxious about interacting with AI. Only if both approach AI, rather than avoid AI, can the human-AI system have the function of providing an objective basis for allocation of scarce healthcare resources. Accordingly, the behavioral ethics of healthcare resource allocation can only be better enabled by a human-AI system if the humans that are involved with the system do not suffer persistent anxiety and stress from interacting with AI. This can depend upon patients and healthcare providers attributing positive intentionality to AI [138,139]. Here, it is important to note that human perception has evolved to facilitate human survival [140,141] and, whatever the visual appearance of AI, patients and healthcare providers may see AI as a threat to survival [82,83,84,85] if they do not attribute positive intentionality to AI. While there may be sufficient time for a healthcare provider to undertake necessary steps to achieve attribution of positive intentionality to AI by its own personnel, such as co-creating working personas and scenarios for the AI [142], it is less likely that there will be enough time to do this for patients.

## 7. Conclusions

Humans no longer have millennia to evolve behavioral traits that can best enable survival in enduring environments. Rather, rapid changes to behaviors and environments come from the introduction of artificial intelligence (AI), and infrastructures that facilitate its application. In this paper, it has been explained how four fundamental questions of behavioral ecology can be applied to inform development of ethical human-AI systems. As summarized in Figure 1, analyzing human-AI systems in terms of function, phylogeny, mechanism, and ontogeny reveals that they can increase ethical stress that can lead to questionable decisions and dubious actions. Accordingly, application of the four fundamental questions can support balanced assessment of ethical human-AI system concepts, and provide a structure to improve their function, phylogeny, mechanism, and ontogeny for behavioral ethics during their development.

Overall, the paper provides two contributions. First, behavioral ecology analysis of behavioral ethics. Second, application of behavioral ecology questions to identify opportunities and challenges for ethical human-AI systems. These include the need for policy-making to encompass the many environmental factors that can affect human-AI systems. This is imperative as there are many scenarios where environmental conditions can lead to situated entropy from system function that can cause ethical stress. Accordingly, it can be appropriate for policy making to be informed through application of methods such as task analysis, and failure mode and effects analysis (FMEA). Task analysis involves detailed evaluation of mental and manual activities in work scenarios and how they can be affected by environmental conditions [143]. FMEA is a risk assessment method that mitigates potential failures in systems, which has been used in a wide range of industries [144].

Human-AI systems may be an important direction for future research into behavioral ethics as more time is spent in environments that are either partially or fully generated by artificial intelligence; for example, in virtual worlds that may be referred to as the metaverse [145,146]. Virtual worlds involve persistent immersive environments within which individual human users can have many avatars [147,148]. Although it is recognized that virtual environments can shape behavior in physical environments [149,150], and that there can be ethical issues from interplay between virtual behavior and physical behavior [151,152], behavioral ethics implications have not been considered in the development of human-AI systems that span physical and virtual environments. For example, via augmented reality [153]. Future research could consider to what extent, if any, human-AI systems can entail complementary physical and virtual sensory ecologies [154,155]. In addition, future research could investigate how to minimize the potential for human-AI systems to introduce perceptual traps [156] and/or ecological traps [157]. In doing so, future research could apply function, phylogeny, mechanism, and ontogeny as structuring constructs to inform the design of ethical human-AI systems that entail human transitions back and forth between physical environments and virtual environments.

## Figures and Tables

**Table 1:** Function: Interrelationships between function fitness, situated entropy, and ethical stress.

Construct		High Fitness	Low Fitness
Situated entropy	Information uncertainty	Low	High e.g., due to truck driver having inadequate route information
	Physical disorder	Low	High e.g., due driving incorrect routes
	Unproductive energy use	Low	High e.g., due to driving incorrect routes
Daily stress	Time pressure	Low	High e.g., no time for work rest breaks that include eating properly
	Self-regulatory depletion	Low	High e.g., from stopping truck at orange traffic lights when late
Chronic stress	Resource loss	Low	High e.g., due to loss of resources because of erratic employment
	Survival anxiety	Low	High e.g., due to employment uncertainty that prevents sleeping well
	Energy imbalance	Low	High, e.g. poor diet and lack of sleep causes allostatic overload
Potential for increased ethical stress		Low	High due to interaction with environment leading to daily stress from high time pressure and high self-regulatory depletion; leading to chronic stress from resource loss, survival anxiety, energy imbalance

**Table 2:** Phylogeny: Interrelationships between trait components and trait robustness.

Behavioral Trait Component	Evolution Span	Current Distribution	Trait Robustness
Human navigation skill	Millennia	Widespread but reducing	Vulnerable to lack of use
Road networks	Centuries	Widespread and increasing	Vulnerable to extreme weather
Trucks	Decades	Widespread and increasing	Vulnerable to extreme weather
Cooperative infrastructure	Years	Very limited but can increase	Vulnerable to extreme weather

**Table 3:** Mechanism: Variables between navigational skill and behavioral ethics.

Mechanism					Behavioral Ethics
Navigation Skill	Infrastructure	Internet	Management Policy	Weather	
High	Cooperative	Reliable	Ethical incentives	Favorable	Low risk
High	Cooperative	Reliable	Ethical incentives	Unfavorable	Low risk
High	Cooperative	Reliable	Productivity incentives	Unfavorable	Medium risk
High	Cooperative	Unreliable	Productivity incentives	Unfavorable	Medium risk
High	Traditional	Unreliable	Productivity incentives	Unfavorable	Medium risk
Low	Cooperative	Reliable	Ethical incentives	Favorable	Low risk
Low	Cooperative	Reliable	Ethical incentives	Unfavorable	Medium risk
Low	Cooperative	Reliable	Productivity incentives	Unfavorable	Medium risk
Low	Cooperative	Unreliable	Productivity incentives	Unfavorable	High risk
Low	Traditional	Unreliable	Productivity incentives	Unfavorable	High risk

**Table 4:** Ontogeny: Effects of background on stress when interacting with AI.

Truck Driver	Background					Ontogeny
	Traditional Navigation Experience	Suspicion of Traditional Navigation	AI-Aided Navigation Experience	Suspicion of AI-Aided Navigation	Propensity for Anxiety	
1	High	None	None	Low	Low	Approaches AI-aided navigation without anxiety
2	High	None	None	High	High	Avoids AI-aided navigation with potential for chronic anxiety
3	Low	Low	High	None	Low	Approaches traditional navigation without anxiety
4	Low	High	High	None	High	Avoids traditional navigation with potential for chronic anxiety

**Table 5:** Vehicle navigation example of opportunities and challenges for behavioral ethics in human-AI systems.

Construct	Opportunities	Challenges
Function	Human-AI truck navigation system can reduce stress-inducing situated entropy experienced by human truck drivers who have poor navigation skills and so could otherwise easily get lost	Continual use of AI-enabled navigation systems can undermine human navigation skills
Phylogeny	Ongoing evolution of technological components has potential to widen the range of human-AI truck navigation systems.	Until there is further evolution of AI components, reduction of stress arising from experience of situated entropy depends upon there being favorable environmental conditions
Mechanism	Human-AI system can include additional components within a management policy that limits the potential for productivity incentives to lead unintentionally to unethical actions	The inclusion of additional components can increase system complexity. Thus, there needs to be system design for high reliability.
Ontogeny	Individualized adaptation of human-AI system to suit individual human truck drivers can be possible	Differences in experience and personality can lead to human interaction with AI leading to unintended ethical stress

**Table 6:** Diagnostic support example of opportunities and challenges for behavioral ethics in human-AI systems.

Construct	Opportunities	Challenges
Function	Reduced situated entropy about basis for treatment decisions, and about allocation of healthcare resources	Reduced situated entropy for patient depends upon patient agreeing with the diagnosis
Phylogeny	Ongoing evolution of technological components has potential to improve diagnoses.	Human-AI system only robust when environment is ideal for gait recording and AI gait analysis is acceptable to the patient
Mechanism	AI-enabled gait analysis has the potential to be seen as providing a diagnosis that is more reliable than that of human healthcare providers alone	AI-enabled gait analysis cannot provide a reliable basis for diagnosis unless many components are combined successfully
Ontogeny	Individualized adaptation of human-AI system to suit individual patients and healthcare providers can be possible	Patient may unintentionally alter gait during gait recording process if has anxiety about interacting with AI-enabled system. Also, human healthcare provider may not trust AI-enabled diagnoses.

## Data Availability

Not applicable.
